# Early type I Interferon response induces upregulation of human β-defensin 1 during acute HIV-1 infection

**DOI:** 10.1371/journal.pone.0173161

**Published:** 2017-03-02

**Authors:** Björn Corleis, Antonella C. Lisanti, Christian Körner, Abigail E. Schiff, Eric S. Rosenberg, Todd M. Allen, Marcus Altfeld, Douglas S. Kwon

**Affiliations:** 1 Ragon Institute of MGH, MIT, and Harvard, Massachusetts General Hospital, Harvard Medical School, Boston, Massachusetts, United States of America; 2 Division of Infectious Diseases, Massachusetts General Hospital, Boston, Massachusetts, United States of America; Centre International de Recherche en Infectiologie (CIRI), FRANCE

## Abstract

HIV-1 is able to evade innate antiviral responses during acute infection to establish a chronic systemic infection which, in the absence of antiretroviral therapy (ART), typically progresses to severe immunodeficiency. Understanding these early innate immune responses against HIV-1 and their mechanisms of failure is relevant to the development of interventions to better prevent HIV-1 transmission. Human beta defensins (HBDs) are antibacterial peptides but have recently also been associated with control of viral replication. HBD1 and 2 are expressed in PBMCs as well as intestinal tissue, but their expression *in vivo* during HIV-1 infection has not been characterized. We demonstrate that during acute HIV-1 infection, HBD1 but not HBD2 is highly upregulated in circulating monocytes but returns to baseline levels during chronic infection. HBD1 expression in monocytes can be induced by HIV-1 *in vit*ro, although direct infection may not entirely account for the increase in HBD1 during acute infection. We provide evidence that HIV-1 triggers antiviral IFN-α responses, which act as a potent inducer of HBD1. Our results show the first characterization of induction of an HBD during acute and chronic viral infection in humans. HBD1 has been reported to have low activity against HIV-1 compared to other defensins, suggesting that *in vivo* induced defensins may not significantly contribute to the robust early antiviral response against HIV-1. These data provide important insight into the *in vivo* kinetics of HBD expression, the mechanism of HBD1 induction by HIV-1, and the role of HBDs in the early innate response to HIV-1 during acute infection.

## Introduction

Human Beta Defensins (HBDs) are a critical part of the early innate immune response against invading pathogens [1–3]. They are short cationic antimicrobial peptides which directly protect against a broad range of bacteria and viruses [3–5]. In addition, they have been shown to induce chemotaxis of T cells, dendritic cells (DCs) and macrophages [4, 6]. Of the 31 genes and pseudogenes that have been identified to encode for beta defensins, only 6 (*HBD1-6*) are known to be expressed [7]. Of these, HBD1-3 have been studied in greatest detail and have detectable protein expression in humans *in vivo* with HBD1 and HBD2 being the only two HBDs expressed in PBMCs as well as mucosal tissue [6–10]. *In vitro* studies have shown antiviral activity of recombinant HBDs against HIV-1 through a variety of mechanisms [11–14]. HBD2 blocks viral entry through direct binding to host entry receptors such as CCR5 and CXCR4 [12–14], as well as induction of chemotaxis of antiviral effector cells [11, 12]. Further, binding of HBD2 to the chemokine receptor CCR6 impairs viral replication by inducing expression of the HIV-1 host restriction factor APOBEC3G (antiviral intrinsic factor apolipoprotein B mRNA editing enzyme, catalytic polypeptide-like 3G) [11, 15–17]. Although HBDs have varying antiviral activity *in vitro*, their expression *in vivo* during HIV-1 infection has not been reported.

Studies have found the highest level of HBDs in tissues, with epithelial cells being the main HBD producing cell type, particularly in mammary glands, urogenital tissue, and the intestinal and respiratory tracts [18]. High expression of HBDs at mucosal surfaces in the gut has been associated with protection against microbial translocation of commensal enteric microbes [19, 20]. Acute HIV-1 infection leads to gut epithelial cell damage and microbial translocation [21, 22] and it has been speculated that epithelial cell damage might lead to downregulation of antibacterial peptides, allowing microbes to cross the mucosal barrier [23]. Some cross sectional studies report HBD1 and 2 production in peripheral blood mononuclear cells (PBMCs) [1, 5], including expression of HBD1 and HBD2 in monocytes, macrophages and DCs [24]. Phagocytes play an important role during the early immune response against HIV-1 and have been shown to be infected and activated during acute HIV-1 infection *in vivo* [25–28]. The ability of HIV-1 to specifically induce or downregulate HBD1 and 2 in phagocytes or epithelial cells is incompletely characterized.

The induction of HBDs by viral and bacterial products suggests involvement of pattern recognition receptors (PRR). The PRR Toll-like receptor 4 (TLR4) has been shown to activate production of HBD2 in epithelial cell lines and DCs after activation with its bacteria-produced ligand, lipopolysaccharide (LPS) [9, 29]. The specific PRR ligands that induce production of HBDs in immune cells such as monocytes and DCs during a viral infection are uncertain. HIV-1 can enter innate immune cells and is sensed by intracellular PRRs [30, 31]. TLR3 and cytosolic dsRNA sensors such as the retinoic acid-inducible gene 1 (RIG-I) receptor [32, 33] recognize viral dsRNA and upregulate type I interferon (IFN) and interferon regulated genes (IRGs) [34–36]. Whether activation of these pathways leads to induction of HBDs has not been investigated.

In this cross-sectional study we characterized the expression of HBD1 and 2 in blood and intestinal tissue during acute and chronic HIV-1 infection. We found that HBD1 is constitutively expressed in epithelial cells in gut tissue and monocytes in the peripheral blood, but upregulated in circulating monocytes during acute HIV-1 infection. HBD2 was not constitutively expressed at baseline levels in gut or peripheral blood and not induced during HIV-1 infection. HIV-1 and IFN-α were both able to induce HBD1 in peripheral monocytes. We show for the first time upregulation of an HBD during an acute viral infection in humans. We provide evidence that HBD1 is part of the early IFN-α antiviral immune response, whereas other HBDs such as HBD2 seem to be part of an antibacterial response. These data improve our understanding of the kinetics and induction of different HBDs in humans in response to viral infections such as HIV-1.

## Material and methods

### Study subjects

Subjects were recruited at Massachusetts General Hospital, Fenway Health Center, and Brigham and Women’s Hospital in accordance with the Partners Institutional Review Board approved protocol (2010P002463). All subjects gave written informed consent for participation in the study. HIV-1 chronic progressors (n = 8) were defined as subjects with plasma HIV-1 RNA viral load (VL) >2000 copies/mL for >12months. HIV-1 viremic controllers (n = 9) maintained plasma VL between <48 copies/ml and <2000 copies/mL for >12 months in the absence of ART. HIV-1 acute infection (n = 32) and categorization into Fiebig stages were defined as described previously [37] and infection with HIV-1 was no longer than 3 months (estimated days of infection). HIV-uninfected control samples (n = 17) were matched to gender and age of the HIV acute group. Detailed clinical and demographic data for each group are listed in S1 Table.

### Human blood and tissue sample

**Fig 1:**
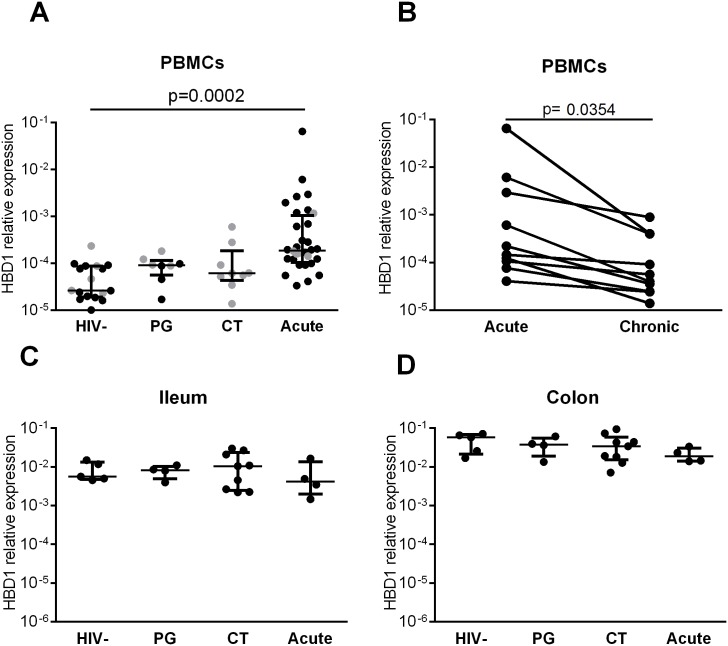
HBD1 is upregulated in PBMCs during acute HIV-1 infection. Samples from HIV-1 uninfected (HIV-), HIV-1 untreated chronic progressors (PG), HIV-1 chronic ART treated (CT) and acutely HIV-1 infected (Acute) individuals were analyzed for HBD1 expression. HBD1 transcription in PBMCs (A, B) or gut pinch biopsies (C, D) was determined by qPCR. Grey color indicates subjects with matched gut biopsy samples. (A) HBD1 expression in blood of acutely infected subjects (n = 32) was found to be significantly higher compared to HIV-1 individuals from PG (n = 8), CT (n = 9) or HIV- (n = 17) subjects (Kruskal-Wallis and Dunn’s multiple comparison test with error bars indicate min and max of boxplots). (B) Longitudinal sampling of individuals (n = 10) showed significant downregulation of HBD1 in chronic HIV-1 infection compared to acute infection (Wilcoxon matched-pairs signed rank test). (C, D) No significant differences in HBD1 expression in ileum (C) or colon (D) was found between HIV- (n = 5), PG (n = 4), CT (n = 9) and HIV acute infected subjects (n = 4), (Kruskal-Wallis and Dunn’s multiple comparison test and median with interquartile range).

Venous blood was collected in acid citrate dextrose tubes and PBMCs were separated by centrifugation on a histopaque gradient and cryopreserved in liquid nitrogen or used fresh. Monocytes were isolated from PBMCs using magnetic-activated cell sorting (MACS) according to the manufacturer’s instructions (Miltenyi). Briefly, cells were incubated with anti-CD14 microbeads for positive selection of CD14+ monocytes using a magnetic column. Bound CD14+ monocytes were washed several times in MACS buffer (PBS + 1% (w/v) FCS) and then collected by elution following removal of the magnetic field. CD14+ monocytes or PBMCs were cultured in R10 (RPMI (Sigma), 10% (v/v) FCS (Sigma), 2 mM Glutamine (Corning), 100 I.U./ml penicillin (Corning) and 100 I.U./ml streptomycin (Corning)) and used for further studies. For sorting of colon tissue and immune cells, macroscopically normal excess surgical colon tissue was received from Massachusetts General Hospital in accordance with an IRB approved protocol (2010P000632). Single cell suspensions from tissue were made by mechanically disrupting dissected tissue with an 18-gauge needle before 20 min incubation at 37°C with 0.5 mg/mL collagenase type II (Clostridium histolyticum, Sigma-Aldrich). The supernatant was filtered through a 70 μm cell strainer and the filtered cells were resuspended in R10+ (R10, 2.5μg/ml amphotericin B, 250 μg/ml piperacillin/tazobactam). This process was repeated once and cells from both filtrations were combined and kept on ice until further analysis. For HBD quantification in bulk gut tissue (Fig 1C and 1D and S1E and S1F Fig), pinch biopsies of intestinal tissue were collected by colonoscopy (transverse colon and terminal ileum) and upper endoscopy (duodenum) for research purposes only and in accordance with a Partners Institutional Review Board approved protocol (2007P002102). Two biopsies from each site were placed immediately into RNAlater (Qiagen) and stored at -80°C until further processing.

### Preparation of HIV-1 stocks

HIV-1 NL4-3 R5 mcherry or NL4-3 R5 GFP virus was used for all *in vitro* experiments and was kindly provided by Thomas Murooka and Thorsten Mempel [38]. HIV-1 was produced and titer determined as described previously [39]. In brief, viral plasmid was transfected into 293T cells using fugene-6 (Polysciences) over night. Cell culture supernatant was removed and replaced with fresh media to avoid harmful side effects of the transfection reagents. Virus was harvested from supernatant 48 h later, aliquoted, and stored at -80°C. Titer of infectious particle (i.p.)/ml was determined by infection of CD4 and CCR5 expressing GHOST cells (NIH Aids Research & Reference Reagent Program) as described previously [39]. A viral stock with a titer of 5x10^6 i.p./ml was used for all experiments. To determine viral entry in monocytes we used the Vpr-BlaM assay as previously described for CD4 T cells [40]. Briefly, NL4-3 R5 pro-viral plasmid DNA was co-transfected with a β-lactamase (BlaM) and the virion protein Vpr coding plasmid (pMM310) into 293T cells to produce a Vpr-BlaM carrying HIV-1 particle.

### Monocyte activation and *in vitro* infection

All cells were incubated for 24 h in R10 media at 37°C and 5% CO_2_. CD14+ cells were stimulated with 5 ug/ml polyinosinic-polycytidylic acid (poly I:C; Invivogen), 200 ng/ml *E*. *coli* lipopolysaccharide (LPS) (Sigma), 2.5 μg/ml Imiquimod (Invivogen), 5 μM ODN2006 (Invivogen), 1 μg/ml 5’ppp-dsRNA (Invivogen) 10–100 pg/ml Interferon alpha (IFN-α) (Reprokine), 5 μg/ml Imiquimod / R837 (Invivogen), 10 pg/ml tumor necrosis factor alpha (TNFα) (Reprokine) or 10 pg/ml interleukin 1 2 beta (IL1-ß) (Reprokine) or 5 μg/ml flagellin from *S*. *typhimurium* (FLA-ST) (Invivogen). Where indicated monocytes were pre-incubated with 50 pg/ml monoclonal anti-IFN-α IgA (Invivogen, #maba-hifna-3) neutralizing antibody or 50 pg/ml IgA control antibody (Invivogen, #maba-2 control) for 30min before prior incubation with 50 pg/ml IFN-α.

For infection of monocytes with HIV-1, 0.5x10^6 CD14+ monocytes were transferred into a 5ml polypropylene tube. The cell pellet was incubated with HIV-1 at a concentration of 1x10^6 i.p. or as indicated in 200 μl R10 at 37°C and 5% CO_2_. After 2h, 800 μl R10+ was added to the tube and cells were further incubated for 24 h at 37°C and 5% CO_2_. Cells were centrifuged for 5 min at 400 x g at 4°C. The cell pellet and the cell culture supernatant were used for further processing or stored at -80°C.

### BlaM-Vpr assay

The BlaM-Vpr assay was used to determine frequency of HIV-1 entry into monocytes. 1x10^5^ CD14+ monocytes were incubated with NL4-3 R5 or X4 BlaM-Vpr virus at a concentration of 250 ng p24 in 200 μl R10 at 37°C and 5% CO_2_ and incubated for 12 h at 37°C and 5% CO_2_. Where indicated, cells were pre-incubated with 40 μM maraviroc (Sigma) prior to infection with HIV-1. Cells were then centrifuged for 5 min at 400 x g at 4°C, washed twice with 1 ml of CO_2_ independent media, and resuspended in 28 μl CCF2-AM loading reagent mix in 150 μl CO_2_ independent media and incubated for 1 h at room temperature (Life Technologies, beta lactamase loading kit). Cells were then washed once with 1 ml CO_2_ independent media and 3 times with 1 ml cold D-PBS with all centrifugation steps for 5 min at 400 x g at 4°C. Cells were resuspended in 100 μl PBS containing 0.1 μl viability stock solution (fixable viability stain 780, BD Bioscience) for 15 min at room temperature. Cells were washed with D-PBS for 5 min at 400 x g at 4°C and resuspended in 200 μl 2% PFA in PBS. Samples were kept in the dark at 4°C before analyzed on a LSR Fortessa flow cytometer (BD Biosciences). Uninfected cells were detected with the violet laser (405 nm, 100mW) and a 450/50 nm bandpass filter whereas infected cells were visible with the violet laser and a 505nm low-pass filter / 525/50 nm bandpass filter. Dead cells were detected with a red laser (640nm, 40mW).

### RNA extraction and PCR

Pinch biopsies of intestinal tissue were mechanically homogenized using a rotostator and then passed over a Qiashredder column (Qiagen). RNA was extracted from homogenized tissue, PBMCs, CD14+ monocytes and sorted cell populations using the RNAeasy kit (Qiagen) following the manufacturer’s protocol. RNA was stored at -80°C until further use or directly transcribed into cDNA using the high capacity cDNA reverse transcriptase kit (Applied Bioscience). HBD1 and HBD2 cDNA was quantified by qPCR using the Brilliant II SYBR^®^ green qPCR master mix (Agilent technology) and analyzed with a MX3000 light cycler (Stratagene) using the following program: Segment 1–95°C for 10:00, Segment 2–95°C for 00:30, 58°C for 01:00, 72°C for 00:30 (run 40 cycles), Segment 3–95°C for 1:00, 55°C for 00:30, 95°C for 00:30. C_t_ values were normalized (ΔC_t_) against the ribosomal housekeeping gene ribosomal protein S9 (RP9) as described and validated elsewhere [41–44]. We did not observe any inflammation or infection induced changes in RP9 C_t_ values. Relative expression of HBD1-2 is presented as the reciprocal log2 of the ΔC_t_ value. Primers were designed using primer-Blast (NCBI; http://www.ncbi.nlm.nih.gov/tools/primer-blast/). All primers spanned at least one exon-exon junction. The following primers were used for all HBD PCR experiments: HBD1F CAGTCGCCATGAGAACTTCCT, HBD1R CACTCCCAGCTCACTTGCAG, HBD2F CCAGCCATCAGCCATGAGGGT, HBD2R GGAGCCCTTTCTGAATCCGCA, HBD3F TCTGCCTTACCATTGGGTT, HBD3R TTTCTTCGGCAGCATTTTC. For quantification of IFNA and ISG15 transcripts, primers were used as published [45, 46].

### HBD1 ELISA

Undiluted cell culture supernatants were loaded onto an ELISA plate coated with antibody against HBD1. ELISA was performed following the manufacturer’s instructions (Peprotech).

### Flow cytometry

Cryopreserved PBMCs from acute (n = 8) and HIV-uninfected control subjects (n = 9) were thawed and 0.5x10^6^ cells were stained for viability with a LIVE/DEAD fixable blue or green dead cell stain kit (Life Technologies). For phenotypic characterization of monocytes and DCs, cell surface antigens were stained for 15 min at room temperature with the following mouse anti-human monoclonal antibodies: FITC anti-CD3 (clone UCHT1; Biolegend), CD19 (clone HIB19; Biolegend), CD56 (clone HCD56; Biolegend), CD66b (clone G10F5; BD Bioscience), BV605 anti-CD4 (clone SK3; BD Bioscience), v500 anti-CD45 (clone HI30; BD Bioscience), APC-H7 anti-CD16 (clone 3G8; BD Bioscience), PE-Cy5 anti-CXCR4 (clone 12G5; BD Bioscience), AF700 anti-HLA-DR (clone L243; BD Bioscience), APC anti-CD11c (clone B-ly6; BD Bioscience), PE-Cy7 anti-CD123 (clone 7G3; BD Bioscience), BV421 anti-CCR5 (clone 2D7; BD Bioscience), PerCP-Cy5.5 anti-CD14 (clone M5E2; Biolegend). The cells were fixed with 2% paraformaldehyde before running on a LSR Fortessa flow cytometer (BD Biosciences) within 4 h. Flow data were analyzed with FlowJo (TreeStar).

### Cell sorting

Single cell suspensions from surgical excess colon tissue or HIV-uninfected control PBMCs were stained for viability with a LIVE/DEAD fixable violet dead cell stain kit (Life Technologies). For phenotypic characterization of epithelial and tissue cells, cell surface antigens were stained as described above with the following mouse anti-human monoclonal antibodies: APC anti-CD326 (clone EBA-1; BD Bioscience), v500 anti-CD45 (clone HI30; BD Bioscience). For phenotypic characterization of monocytes and DCs from PBMCs, cell surface antigens were stained for 15 min at room temperature with the following mouse anti-human monoclonal antibodies: FITC anti-CD3 (clone UCHT1; Biolegend), CD19 (clone HIB19; Biolegend), CD66b (clone G10F5; BD Bioscience), v500 anti-CD45 (clone HI30; BD Bioscience), APC-H7 anti-CD16 (clone 3G8; BD Bioscience), AF700 anti-HLA-DR (clone L243; BD Bioscience), APC anti-CD11c (clone B-ly6; BD Bioscience), PE-Cy7 anti-CD123 (clone 7G3; BD Bioscience), PerCP-Cy5.5 anti-CD14 (clone M5E2; Biolegend). Stained cells were resuspended in PBS with 5 mM EDTA and sorted using a FACS Aria II (BD Biosciences).

### Statistical analysis

Nonparametric tests were used to compare medians between groups. The Mann-Whitney test was used for 2 groups and the Kruskal-Wallis test followed by Dunn’s multiple comparison post tests for >2 groups. Differences were considered significant at p < 0.05. Graphpad Prism 6 was used for all analyses.

## Results

### Human beta defensin 1 is upregulated during acute HIV-1 infection

HBD 1 and 2 are the only human beta defensins which have been reported to be expressed in both peripheral blood and mucosal tissue [24, 29, 47, 48]. Human beta defensins have shown potent antiviral activity against several viruses, including HIV-1 [1, 6, 11, 12, 18, 27, 49, 50]. However, the temporal dynamics of expression of HBDs during HIV-1 infection remains uncharacterized. We investigated transcriptional levels of HBD1 and 2 using PBMCs and intestinal pinch biopsies from individuals at different stages of HIV-1 infection. HBD1 was constitutively expressed in PBMCs obtained from HIV-1 uninfected subjects with levels significantly increased during acute HIV-1 infection (4-fold increase relative to HIV-1 uninfected, p = 0.0002) but not in those with chronic progressive (PG) or immunologically controlled (CT) infection (Fig 1A). Longitudinal sampling of individuals progressing from acute to chronic infection revealed that HBD1 was down-regulated in the same individuals at later stages of untreated infection (Fig 1B, p = 0.0354). In contrast to HBD1, HBD2 was undetectable or expressed at low levels in PBMCs, regardless of HIV status (S1 Fig). Notably, transcription of the antiviral peptide HBD3, was not detected in any intestinal or blood sample by qPCR (data not shown). Our data indicate that HBD1 is constitutively expressed in PBMCs and is upregulated during acute HIV-1 infection but returns to baseline levels during chronic progressive infection. Further, the antiviral peptides HBD2 or HBD3 were not induced during acute HIV-1 infection.

HBDs have been reported to be primarily expressed in epithelial cells of the gut mucosa [1, 18, 51]. We therefore investigated whether transcription of HBD1 was also altered during HIV-1 infection in intestinal tissue. HBD1 was found to be constitutively transcribed at high levels in ileum and colon obtained by gut biopsies from healthy individuals (Fig 1C and 1D). However, transcription levels were not altered during acute or chronic HIV-1 infection (Fig 1C and 1D). Although the number of gut biopsy samples from acute infected subjects was low (n = 4), cross-sectional analysis between matched PBMCs and intestinal samples from the same individuals revealed that HBD1 was significantly increased in PBMCs but not in colon or ileum relative to HIV-1 uninfected subjects (S1 Fig), p = 0.0015). Similar to our findings in PBMCs, HBD2 transcription in gut tissue was only found in a few samples across different cohorts, with low expression levels and no induction in HIV-1 positive samples detected (S1 Fig). Similarly, we were not able to detect HBD3 transcription in intestinal tissue samples from our cohort (data not shown). These findings demonstrate that upregulation of HBD1 during acute HIV-1 infection appeared to be compartment specific and that intestinal transcription of HBDs during HIV-1 infection is not altered.

**Fig 2:**
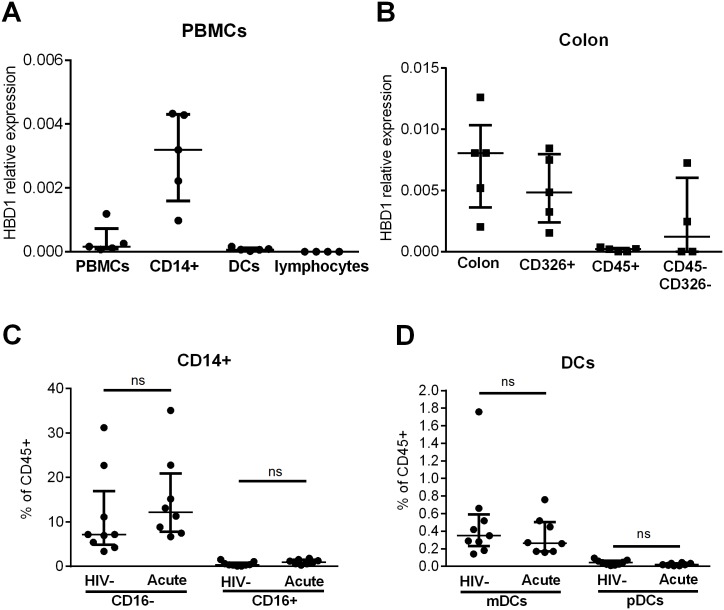
HBD1 is expressed by circulating monocytes and gut epithelial cells. (A, B) Cells were sorted from 5 different bulk colon excess tissue (n = 5) or PBMCs (n = 5), RNA extracted and HBD1 transcription quantified by qPCR. For colon, epithelial cells were identified using the surface marker CD326 and CD45 (S2 Fig). For PBMCs, monocytes were identified as CD14+, DCs as CD11c+ (mDCs) or CD123+ (pDCs) and lymphocytes as CD3+CD19+ (S2 Fig). Epithelial cells were the main producers of HBD1 in colon whereas CD14+ cells were the main producers in PBMCs. (C, D) The frequency of HBD1 producing cells is not altered during acute infection. Frequency of monocytes (C) and DC (D) populations in PMBCs from HIV-1- (n = 9) or acutely (Acute; n = 8) infected individuals was determined by flow cytometry. Conventional monocytes were identified as CD14+ CD16-, inflammatory monocytes as CD14+ CD16+ and mDCs as well as pDCs as described above (S2 Fig). No significant difference in cell frequencies was found between HIV-1- and sample from subjects with acute HIV-1 infection for any of the analyzed cell populations (Kruskal-Wallis and Dunn’s multiple post comparison test). (A-D) Data points are presented with median and interquartile range.

To determine the specific cell lineages responsible for HBD1 expression in blood in comparison to intestinal tissue, we sorted blood CD14+ monocytes, DCs (mDCs and pDCs combined) and lymphocytes (CD19+ B cells and CD3+ T cells combined). For colon tissue, we sorted intestinal epithelial cells (CD326+), tissue resident leukocytes (CD45+CD326-) and stromal tissue cells (CD326-CD45-) (S2 Fig). Overall, transcription of HBD1 was higher in colon compared to PBMCs (Fig 2A and 2B). In PBMCs, CD14+ monocytes (CD16+ and CD16-) were the main cell type expressing HBD1 transcripts, with some expression in DCs and no detectable transcription in lymphocytes (Fig 2A). As expected, the highest transcription in colon was found in epithelial cells, followed by tissue stromal cells, and there were low but detectable levels in CD45+ tissue immune cells (Fig 2B). These results suggest that in colon, HBD1 was constitutively produced by epithelial and tissue cells whereas in PBMCs, CD14+ monocytes were the main HBD1 producers.

To assess whether alterations in cell frequencies during acute HIV-1 infection might impact our results, we compared the frequency of conventional (CD14+CD16-) and inflammatory monocytes (CD14+CD16+) as well as mDCs (CD11c+HLADR+) and pDCs (HLADR+CD123+) in PBMCs from acutely infected subjects and HIV-1 uninfected control subjects. No significant differences were found when comparing the frequency of these cell types, suggesting that increased level of HBD1 was not due to an increase in frequency of HBD1 producing cells (Fig 2C and 2D and S2 Fig) but rather due to upregulation of HBD1 in monocytes.

### HIV-1-induced upregulation of HBD1

**Fig 3:**
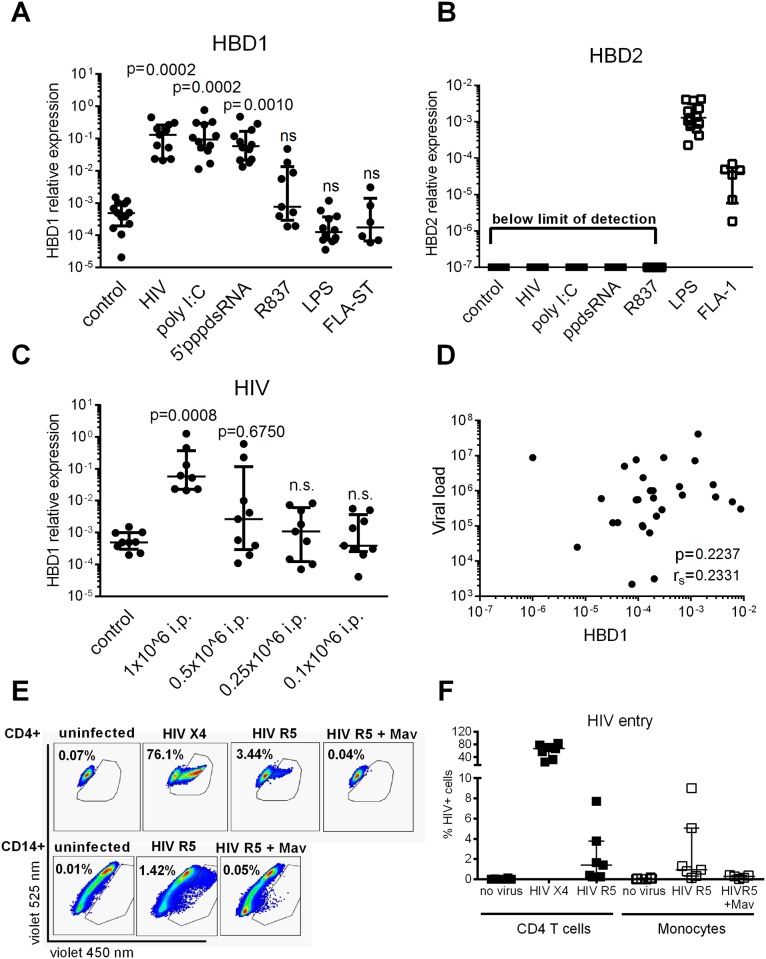
HIV-1 induces HBD1 *in vitro* but is unlikely to be sufficient *in vivo*. (A-C) CD14+ monocytes isolated using Miltenyi MACS technology were incubated with either HIV-1 R5, poly I:C (TLR3 ligand), 5’ppp-dsRNA (RIG-I ligand), R837 (TLR7 ligand), LPS (TLR4 ligand) or FLA-ST (TLR5 ligand), or left untreated. After 24 h cells were harvested and RNA extracted. Relative expression of HBD1 (A, C) or HBD2 (B) was assessed using quantitative PCR. HBD1 expression was found to be significantly increased in monocytes when incubated with HIV-1 R5, poly I:C or 5’pppdsRNA (A), whereas LPS and FLA-ST induced HBD2 (B). (C) Significant upregulation of HBD1 by HIV-1 was only achieved at 1x10^6 infection HIV-1 particles (i.p.) (Kruskal-Wallis and Dunn’s multiple post comparison test). Data points are presented with median and interquartile range which each dot representing an independent experiment from a different healthy control subject. (D) HBD1 transcription in PBMCs of subjects with acute HIV-1 infection (Fig 1A) was plotted against the corresponding viral load which each dot representing one subject. No significant correlation was found between viral load and HBD1 transcription (Spearman r correlation). (E, F) CD4+ T cells or CD14+ monocytes were isolated from PBMCs and incubated with either β-lactamase packaged HIV-1 X4 (CXCR4 tropic)/ HIV-1 R5 (CCR5 tropic) or left untreated. Where indicated cells were pre-treated with the CCR5 antagonist maraviroc (Mav) for 30min before HIV-1 treatment. All virus contained Vpr-BlaM which allowed assessment of HIV-1 entry into cells 12h after exposure. The highest entry was observed in CD4+ T cells by HIV-1 X4, whereas HIV-1 R5 entered CD4+ T cells as well CD14+ monocytes at low frequency. The graph shows the median and interquartile range with each dot representing one independent experiment from a different healthy control subject (F).

We next investigated whether HIV-1 directly induced HBD1 transcription. Incubation of CD14+ monocytes with R5-tropic HIV-1 significantly increased transcription of HBD1 (Fig 3A, p = 0.0002). HIV-1 induced HBD1 transcription was inhibited when incubating monocytes with the CCR5 antagonist maraviroc (mav), suggesting that viral entry was required for HDB1 transcription (S2 Fig). Further, both the TLR3 ligand poly I:C and the RIG-I ligand 5’ppp-dsRNA significantly upregulated HBD1 transcription, whereas exposure of monocytes to the TLR7 ligand R837 (Fig 3A), the TLR9 ligand ODN2006 (S3A Fig) or bacterial products such as the TLR4 ligand LPS or the TLR5 ligand FLA-ST did not increase HBD1 transcription (Fig 3A). Interestingly, LPS or FLA-ST induced HBD2 transcription in CD14+ monocytes (Fig 3B), suggesting that HBD1 is induced by viral products whereas HBD2 is induced by bacterial products.

Although HIV-1 was sufficient to induce HBD1 production *in vitro*, this required high amounts of HIV-1. We therefore assessed whether HIV-1 mediated HBD1 induction could occur when incubating monocytes with concentrations of HIV-1 in the range of the detected plasma viral loads of subjects in the acute HIV-1 group (S1 Table). Concentrations of HIV-1 below 1x10^6^ infectious particle / ml did not induce significant upregulation of HBD1 in monocytes *in vitro* (Fig 3C). Further, HBD1 transcription did not correlate with HIV-1 plasma viral load *in vivo*. Next, we investigated whether HIV-1 R5 enters monocytes and therefore triggers HBD1 transcription directly. We made use of the previously described Vpr-BLaM assay to detect frequency of early viral entry [40]. Non-activated CD4+ T cells and CD14+ monocytes were isolated from PBMCs obtained from healthy subjects and infected with either X4 or R5-tropic HIV-1. CD4 T cells served as a positive control. After 12 h, the majority of control CD4 T cells showed high levels of entry of X4 but not R5-tropic HIV-1 (Fig 3E and 3F). Efficient X4 tropic virus entry was expected since CD4 T cells express high levels of CXCR4. CCR5 mediated entry into CD4 T cells was specific since the CCR5 antagonist maraviroc was able to block infection. Although we used a high viral titer, only 1–3% of monocytes were permissive to HIV-1 entry (Fig 3E and 3F). Together, these data suggest that HIV-1 as well as ligands for TLR3 and RIG-I can directly induce HBD1 transcription. However, monocytes are not permissive to efficient HIV-1 entry and physiological viral titers may not be sufficient to account entirely for HBD1 induction observed during acute HIV-1 infection.

### HBD1 is part of the early IFN-α induced antiviral immune response

**Fig 4:**
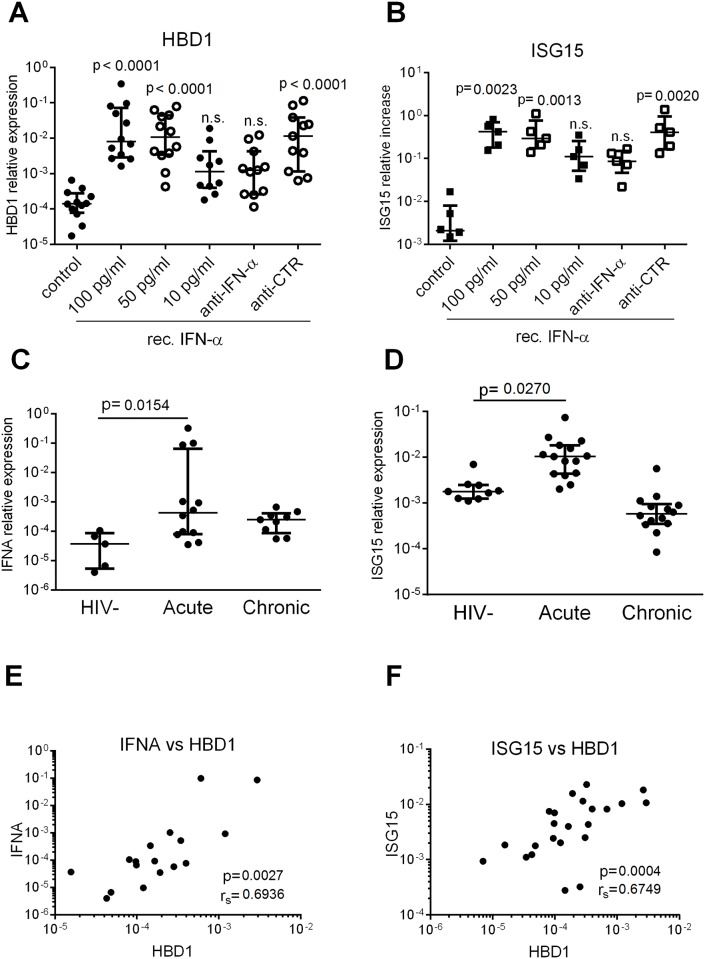
IFN-α is a potent inducer of HBD1 in monocytes *in vitro* and correlates with HBD1 transcription *in vivo*. (A, B) CD14+ monocytes (A n = 11 and B n = 5) were isolated from PBMCs and incubated with different concentrations of recombinant IFN-α2 or left untreated. Where indicated cells were treated with either an anti-IFN-α or an isotype control antibody 30 min before stimulation with 50 pg/ml recombinant IFN-α2. Relative expression of HBD1 and ISG15 was assessed using quantitative PCR. IFN-α2 was found to significantly upregulate HBD1 and ISG15. Each dot represents one independent experiment from a different healthy control subject. (C, D) Human PBMCs were isolated from whole blood from HIV-1 uninfected (HIV-) (C n = 5, D n = 9), HIV-1 untreated chronic progressors (PG) (C n = 9, D n = 14) and acutely HIV-1 infected (Acute) individuals (C n = 12, D n = 15). IFNα (IFNA) transcription of PBMCs was assessed by qPCR. IFNA and ISG15 were both significantly upregulated in acutely but not chronically infected subjects compared to HIV-uninfected control subjects (A-D) Kruskal-Wallis and Dunn’s multiple comparison test with graphs showing median and interquartile range. (E, F) HBD1 transcription in PBMCs of subjects with acute HIV-1 infection (Fig 1A) was plotted against the corresponding transcription of IFNA (E) or ISG15 (F) with each dot representing one subject. ISG15 and IFNA were found to significantly correlate with HBD1 transcription in acutely infected individuals (Spearman r correlation).

IFN-α is a key regulator of genes with anti-HIV-1 activity during acute infection [52]. We therefore tested whether IFN-α induced upregulation of HBD1 as part of the early interferon stimulated innate immune response against HIV-1 [53]. Incubation of monocytes with physiologically relevant concentrations of recombinant IFN-α was associated with significant (p< 0.0001) upregulation of HBD1 transcription (Fig 4A). Upregulation of HBD1 was dependent on IFN-α concentration and blocked by an anti-IFN-α IgA neutralizing but not control IgA antibody (Fig 4A). Incubation with recombinant IFN-α as well as HIV-1 or poly I:C led not only to induction of HBD1 transcripts but also to release of HBD1 peptide as measured by ELISA in cell culture supernatants (S2 Fig). Notably, in the same conditions recombinant IFN-α also significantly upregulated other well-known interferon stimulated genes such as ISG15 (Fig 4B), suggesting that IFN-α could potentially contribute to HIV-1 mediated upregulation of HBD1 *in vivo* as part of the early innate immune response. To analyze this further, we determined the level of *IFNA* and *ISG15* transcripts in PBMCs from the acute patient cohort. We found *IFNA* and *ISG15* transcripts upregulated in acute PBMCs compared to HIV-uninfected control subjects and HIV chronic progressors. HIV-1 induces also other pro-inflammatory cytokines during acute infection such as TNF-α and IL1-β [53–55], however these cytokines did not induce HBD1 transcription in monocytes (S3B Fig). Importantly, *IFNA* as well as *ISG15* transcriptional level correlated strongly with HBD1 transcripts supporting the finding that HBD1 is induced by IFN-α rather than HIV-1 directly. Together, these data suggest that although HIV-1 can directly induce HBD1 expression, HIV-1-induced type I interferons may play a role in HBD1 upregulation as part of the early innate immune response during acute HIV-1 infection.

## Discussion

Failure of early innate effector mechanisms allows HIV-1 to establish a chronic lifelong infection. Understanding these early responses is therefore important to improve strategies for protecting against HIV-1 transmission. In this study, we characterized HBD expression *in vivo* during early and chronic HIV-1 infection. We report that among the HBDs, only HBD1 is upregulated in circulating monocytes during acute HIV-1 infection and that this increase is likely mediated as part of the early type I interferon innate antiviral immune response. Although HBDs have been described as having antiviral activity *in vitro*, this is the first report showing that HBD1 is highly upregulated during acute HIV-1 infection *in vivo*. Importantly, *in vitro* HBD2 and HBD3 showed strong antiviral activity whereas HBD1 showed no significant antiviral activity against HIV-1 [11, 12, 27, 50]. These studies indicate that *in vivo* HBD1 may not have the same impact as HBD2 and HBD3 on HIV-1 transmission and viral control. Therefore, IFN-α induced upregulation of HBD1 may not support type I interferon induced antiviral defense mechanisms.

We report that HBD1 upregulation *in vivo* is triggered by IFN-α. However, *in vitro* HIV-1 infection of monocytes using high viral amounts induces similar levels of HBD1 transcripts. We observed direct induction of HBD1 by HIV-1 but this is unlikely to be the only mechanism of HBD1 upregulation given that HBD1 transcription returned to baseline during chronic HIV-1 infection despite high plasma viral load. A limitation of the study is the small sample size in the group of HIV-infected progressors (Fig 1A), which may explain why we did not observe significant upregulation of IFNA and ISG15 during the chronic phase of HIV-1 infection as reported in other studies [56]. However we found statistically significant downregulation of HBD1 when following acutely infected subjects over time (Fig 1B), supporting our conclusion that HBD1 is not upregulated during chronic HIV-1 infection. Additionally, HBD1 did not correlate with plasma viral loads during acute or chronic infection and the frequency of infected monocytes was below 5% *in vitro* and may not solely explain the significant upregulation of HBD1 *in vivo*. HIV-1 entry was required for HBD1 upregulation since the CCR5 antagonist maraviroc blocked HIV-1-induced HBD1 upregulation, suggesting that sensing of intracellular HIV-1 was necessary for this induction. We also showed that increased production of HBD1 may be mediated through TLR3 and RIG-I ligands. Interestingly, TLR7 activation (Fig 3) or TLR9 activation (S3A Fig) did not result in HBD1 upregulation. It has been reported that RIG-I can be activated by HIV-1 viral RNA *in vitro* and that RIG-I can induce interferons as well as the HIV host restriction factor APOBEC-3 [32, 33]. TLR3 has been described to induce antiviral activity during acute HIV-1 infection [35, 57], including upregulation of antiviral micro-RNAs in macrophages [57]. The impact of RIG-I on HIV-1 mediated HBD1 transcription is likely higher than that of TLR3 since HIV-1 RNA after viral entry is located in the cytoplasm rather than in the endosome. Activation of RNA sensing receptors by HIV-1 could activate not only HBD1 but also additional antiviral pathways leading to either protection or pathology. It will be of interest to study further whether HIV-1 infection truly results in activation of RIG-1 and TLR3 signaling in monocytes *in vivo* and whether this has an impact on viremia and disease progression.

Other cell types such as pDCs have also been described to express HBD1 [58–60], however we found only low constitutive transcription of HBD1 in DCs and little upregulation of HBD1 after stimulation (data not shown). HBD1 expression levels were 10–50 times lower in DCs compared to monocytes and are therefore unlikely to significantly contribute to increased HBD1 levels in PBMCs *in vivo*. However, it is possible that DCs indirectly stimulate production of HBD1 observed during acute HIV-1 infection by secretion of IFN-α. In fact, pDCs are thought to be the main source of plasma IFN-α during viral infections and low frequencies of pDCs are sufficient to produce enough IFN-α to stimulate co-cultured monocytes [61]. Platelets have been shown to be another potential source of HBD1 as they have been shown to store HBD1 in lipid vesicles and release it upon stimulation [60]. We did not investigate release of HBD1 from platelets, however HBD1 release by platelets has only been reported after incubation with bacteria [60] and we found HBD1 release dependent on viral RNA recognition. Additionally, IFN receptors are not expressed by platelets [59].

Previous reports demonstrated high levels of HBD expression in epithelial cells of tissue compartments such as the intestinal tract. Transcription of HBD1 was 10–100 times higher in gut tissue compared to PBMCs. However, transcription was not altered in this compartment during acute infection. We found HBD1 transcription to be induced directly by HIV-1 and by IFN-α. HIV-1 is not known to infect epithelial cells efficiently and therefore would not be expected to reach the intracellular compartment in high amounts. In contrast, type I interferon receptors are expressed by all nucleated cell types [62]. However, parenteral administration of type I interferons in mice does not lead to epithelial cell activation due to the apical orientation of the IFN-α/β receptors [63]. Therefore, HIV-1 induced expression of type I interferons on the basolateral side might not lead to HBD1 upregulation in epithelial cells. This does not exclude the possibility that gut-resident DCs produce IFN-α upon HIV-1 induced intestinal inflammation [64]. We were not able to measure IFNA transcripts in gut biopsy tissues due to limitations in sample material. It would be important to correlate IFNA with HDB1 transcription in this compartment to fully understand the role of type 1 interferons in the regulation of defensins in the gut. Other regulatory pathways have been described to upregulate HBD1 in intestinal epithelial cells, such as hypoxia inducible factor-1 alpha (HIF-1α) [7, 65], the transcription factor c-Myc, or the circadian clock protein BMAL1 [7]. HIF-1α and c-Myc control HBD1 expression in epithelial cells in a non-inflammatory manner [7], supporting our findings that HBD1 transcription is not altered during acute viral infection in these cells. Acute HIV-1 also did not lead to downregulation of HBD1 in the intestinal tract (S2 Fig) suggesting that microbial translocation is not linked to a lack of HBD expression. However, we have not measured protein levels in this compartment and our sample size was relatively small (n = 4). Indeed, we noted a trend towards lower HBD1 transcription in colon from subjects with acute HIV-1 infection compared to controls. This trend might reach significance with a larger sample size. It will be interesting to see whether larger cohorts would be able to identify small differences in HBD1 transcription in gut epithelial cells during HIV-1 infection.

In our study, the two beta defensins with reported anti-HIV1 activity, HBD2 and HBD3 were not induced during acute or chronic HIV-1 infection in PBMCs or gut tissue. However, we observed increased transcription of HBD2 by LPS or flagellin in blood monocytes, consistent with previous findings that bacterial products induce HBD2 expression in intestinal epithelial cells and PBMCs [66, 67]. Furthermore, we observed a trend toward lower HBD1 transcription in LPS-stimulated PBMCs (Fig 3A). These observations suggest HBD1 and HBD2 are induced by viral or bacterial products respectively, and regulated by distinct pathways. It remains uncertain whether induction of HDB2 expression *in vivo* would be beneficial and reduce viral burden. However, induction of this antiviral peptide by bacterial products such as LPS may exacerbate the chronic inflammation and inflammation related diseases observed in HIV-1 infected individuals [68].

The impact of HBD1 on HIV-1 acquisition and progression has been studied on a population level. Three studies investigated polymorphism in the *DEFB1* gene and their impact on mother-to-child HIV-1 transmission. Two studies found an increase of a common polymorphism in infected versus uninfected children [69, 70] and one study showed a correlation of polymorphisms with HIV-1 copies in the breast milk of infected woman [71]. A more recent study in a cohort of HIV-1 infected adults off ART associated polymorphisms in *DEFB1* with viral load and CD4 counts [72]. However, these studies did not address whether HIV disease markers correlated with increased or decreased protein levels of HBD1. Overall, our study provides new insights into the kinetics of HBD1 expression *in vivo* during acute HIV-1 infection and identifies a specific mechanism by which HIV-1 infection results in upregulation of HBD1. This work contributes to our understanding of the role of defensins in early innate antiviral responses, HIV-1 evasion strategies, and may lead to the development of interventions to prevent and control HIV-1 transmission and disease progression.

## Supporting information

S1 Fig HBD2 and HBD1 transcription in intestinal tissue is not altered during HIV-1 infection. (A) Human PBMCs were isolated from whole blood from HIV-1 neg (n = 6), HIV-1 untreated chronic progressors (PG; n = 8), HIV-1 controllers (CT; n = 9) and acutely HIV-1 infected (Acute; n = 32) individuals. HBD2 and HBD1 transcription was assessed by qPCR. (B) RNA was extracted from gut pinch biopsies from HIV-1- (n = 5), untreated chronic progressors (PG; n = 4), HIV-1 controller (CT; n = 9) and HIV-1 acutely (Acute; n = 4) infected individuals and HBD2 transcription was assessed by qPCR. No statistical test was performed as only 11 samples from all groups and compartments had detectable levels of HBD2. (D-F) No significant differences in HBD1 expression in colon (D) or ileum (E) were found between HIV-uninfected and HIV acute infected subjects, whereas the same subjects showed a significant increase of HBD1 in blood (F) (Mann-Whitney test with graphs showing median and interquartile range).
(TIF)

S2 Fig Gating strategy for flow cytometry and HIV-1 induced HBD1 transcription and peptide release. For sorting of epithelial cells and CD45+ cells (Fig 2B) from colon tissue, epithelial cells were identified by gating on cells in the SSC-A/FSC-A, viable cells, CD45- and CD326 high. Gut resident leukocytes were identified as CD45+ and tissue fibroblasts as CD326-CD45-. (B) For cell sorting (Fig 2A) or flow cytometry analysis (Fig 2C and 2D) of PBMCs, cells were gated by SSC/FSC, viability and CD45+. Lymphocytes were identified as CD19 (B cells), CD3 (T cells) and CD 56 (NK cells) positive. Monocytes were identified as CD3-CD19-CD56-CD14+. For analysis of monocyte subsets pro-inflammatory monocytes were identified as CD14+CD16+ and classical monocytes as CD14+CD16-. DCs populations were identified as CD3-CD19-CD56-HLADR+CD14-and CD11c+ (mDCs) or CD3-CD19-CD56-HLADR+CD14-CD11c-CD123+ (pDCs). For cell sorting mDCs and pDCs were pooled into one DCs population (Fig 2A). (C) CD14+ monocytes (n = 12) isolated using Miltenyi MACS technology were incubated with either HIV-1 R5 or left untreated. Where indicated, cells were pre-incubated with the CCR5 antagonist maraviroc (mav) 30 min prior to exposure to HIV-1. After 24 h cells were harvested and RNA extracted. Relative expression of HBD1 was found to be significantly increased in monocytes when incubated with HIV-1 R5 which was inhibited by maraviroc treatment. (D) Release of HBD1 peptide was determined in cell culture supernatants by ELISA. Release of HBD1 peptide was significantly induced by stimulation of monocytes (n = 6) with high concentrations of HIV-1, IFN-α or the TLR3 ligand poly I:C compared to untreated control cells. Kruskal-Wallis and Dunn’s multiple comparison test with graphs showing median and interquartile range and each dot representing one independent experiment from a different healthy control subject.
(TIF)

S3 Fig The TLR9 agonist ODN2006 and the pro-inflammatory cytokines TNF-α or IL1-β do not induce HBD1 transcription in monocytes. (A-C) CD14+ monocytes (n = 4) isolated using Miltenyi MACS technology were incubated with 5μM (TLR9 ligand), 10pg/ml TNF-α, 10pg/ml IL1-β or left untreated. After 24 h cells were harvested and RNA extracted. Relative expression of HBD1 was assessed using quantitative PCR. HBD1 expression was not significantly increased in monocytes when incubated with the TLR9 ligand, TNF- α or IL1-β ((A) Mann-Whitney test, (B) Kruskal-Wallis and Dunn’s multiple post comparison test). Data points are presented with median and interquartile range which each dot representing an independent experiment from a different healthy control subject.
(TIF)

S1 Table Clinical and demographic data of HIV-1 infected and HIV-1 uninfected subjects. (DOCX)
