# Supplementary figures and images for: Early type I Interferon response induces upregulation of human β-defensin 1 during acute HIV-1 infection
Source: PLoS One. 2017 Mar 2;12(3):e0173161. doi: 10.1371/journal.pone.0173161 (PMC5333889; doi:10.1371/journal.pone.0173161)

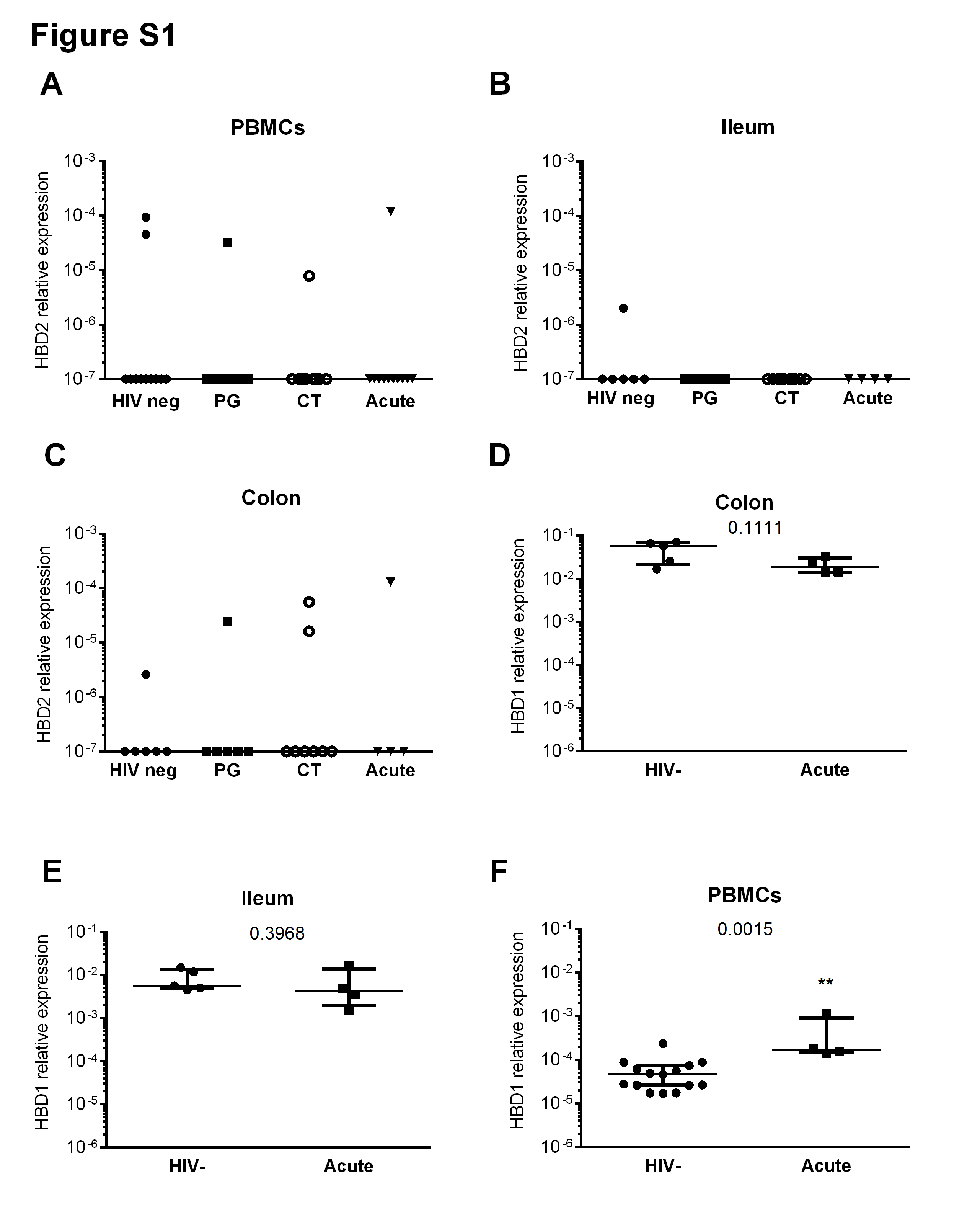

Supplement: S1 Fig — (A) Human PBMCs were isolated from whole blood from HIV-1 neg (n = 6), HIV-1 untreated chronic progressors (PG; n = 8), HIV-1 controllers (CT; n = 9) and acutely HIV-1 infected (Acute; n = 32) individuals. HBD2 and HBD1 transcription was assessed by qPCR. (B) RNA was extracted from gut pinch biopsies from HIV-1- (n = 5), untreated chronic progressors (PG; n = 4), HIV-1 controller (CT; n = 9) and HIV-1 acutely (Acute; n = 4) infected individuals and HBD2 transcription was assessed by qPCR. No statistical test was performed as only 11 samples from all groups and compartments had detectable levels of HBD2. (D-F) No significant differences in HBD1 expression in colon (D) or ileum (E) were found between HIV-uninfected and HIV acute infected subjects, whereas the same subjects showed a significant increase of HBD1 in blood (F) (Mann-Whitney test with graphs showing median and interquartile range). (TIF) [file pone.0173161.s001.tif]

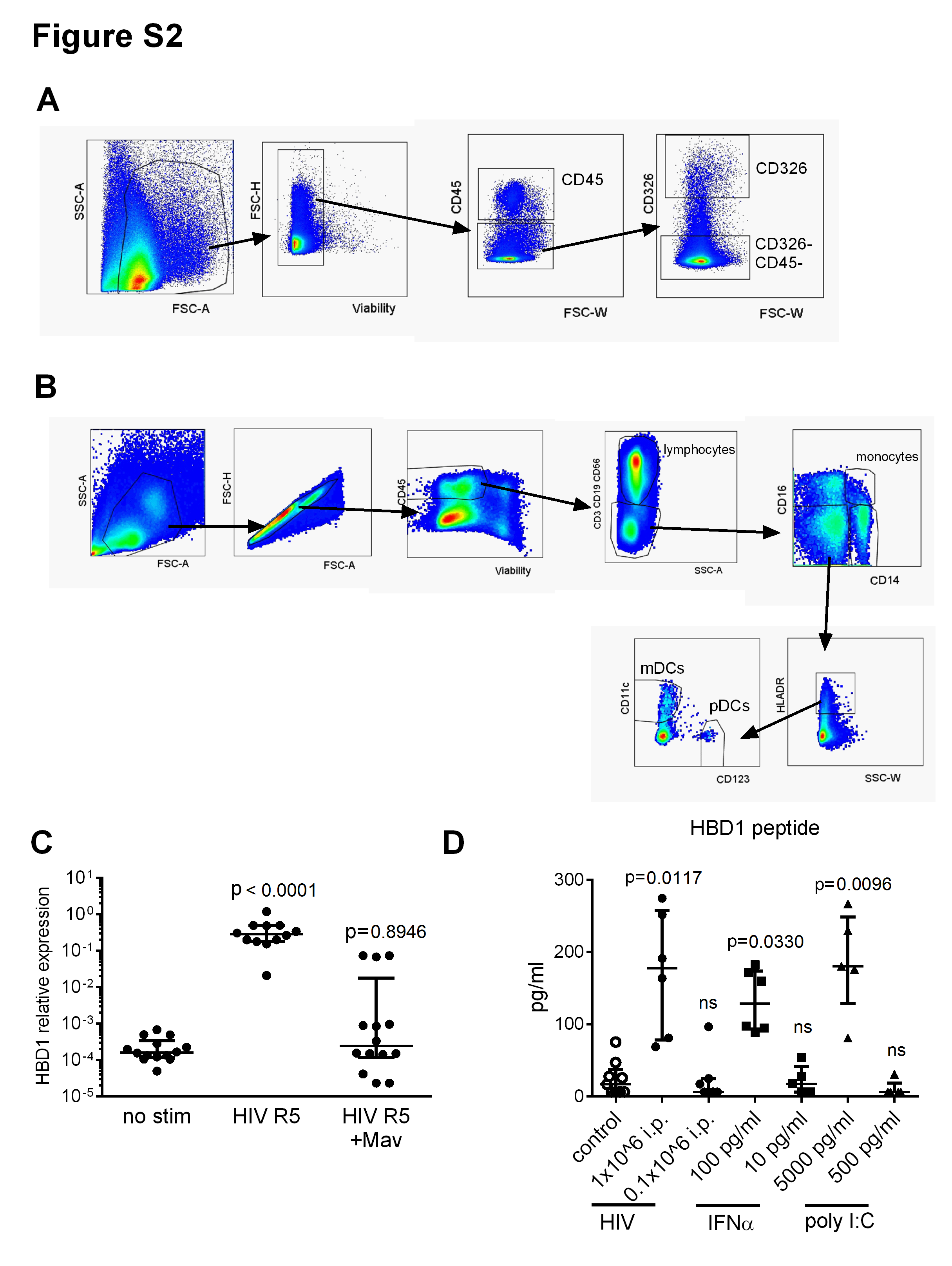

Supplement: S2 Fig — For sorting of epithelial cells and CD45+ cells (Fig 2B) from colon tissue, epithelial cells were identified by gating on cells in the SSC-A/FSC-A, viable cells, CD45- and CD326 high. Gut resident leukocytes were identified as CD45+ and tissue fibroblasts as CD326-CD45-. (B) For cell sorting (Fig 2A) or flow cytometry analysis (Fig 2C and 2D) of PBMCs, cells were gated by SSC/FSC, viability and CD45+. Lymphocytes were identified as CD19 (B cells), CD3 (T cells) and CD 56 (NK cells) positive. Monocytes were identified as CD3-CD19-CD56-CD14+. For analysis of monocyte subsets pro-inflammatory monocytes were identified as CD14+CD16+ and classical monocytes as CD14+CD16-. DCs populations were identified as CD3-CD19-CD56-HLADR+CD14-and CD11c+ (mDCs) or CD3-CD19-CD56-HLADR+CD14-CD11c-CD123+ (pDCs). For cell sorting mDCs and pDCs were pooled into one DCs population (Fig 2A). (C) CD14+ monocytes (n = 12) isolated using Miltenyi MACS technology were incubated with either HIV-1 R5 or left untreated. Where indicated, cells were pre-incubated with the CCR5 antagonist maraviroc (mav) 30 min prior to exposure to HIV-1. After 24 h cells were harvested and RNA extracted. Relative expression of HBD1 was found to be significantly increased in monocytes when incubated with HIV-1 R5 which was inhibited by maraviroc treatment. (D) Release of HBD1 peptide was determined in cell culture supernatants by ELISA. Release of HBD1 peptide was significantly induced by stimulation of monocytes (n = 6) with high concentrations of HIV-1, IFN-α or the TLR3 ligand poly I:C compared to untreated control cells. Kruskal-Wallis and Dunn’s multiple comparison test with graphs showing median and interquartile range and each dot representing one independent experiment from a different healthy control subject. (TIF) [file pone.0173161.s002.tif]

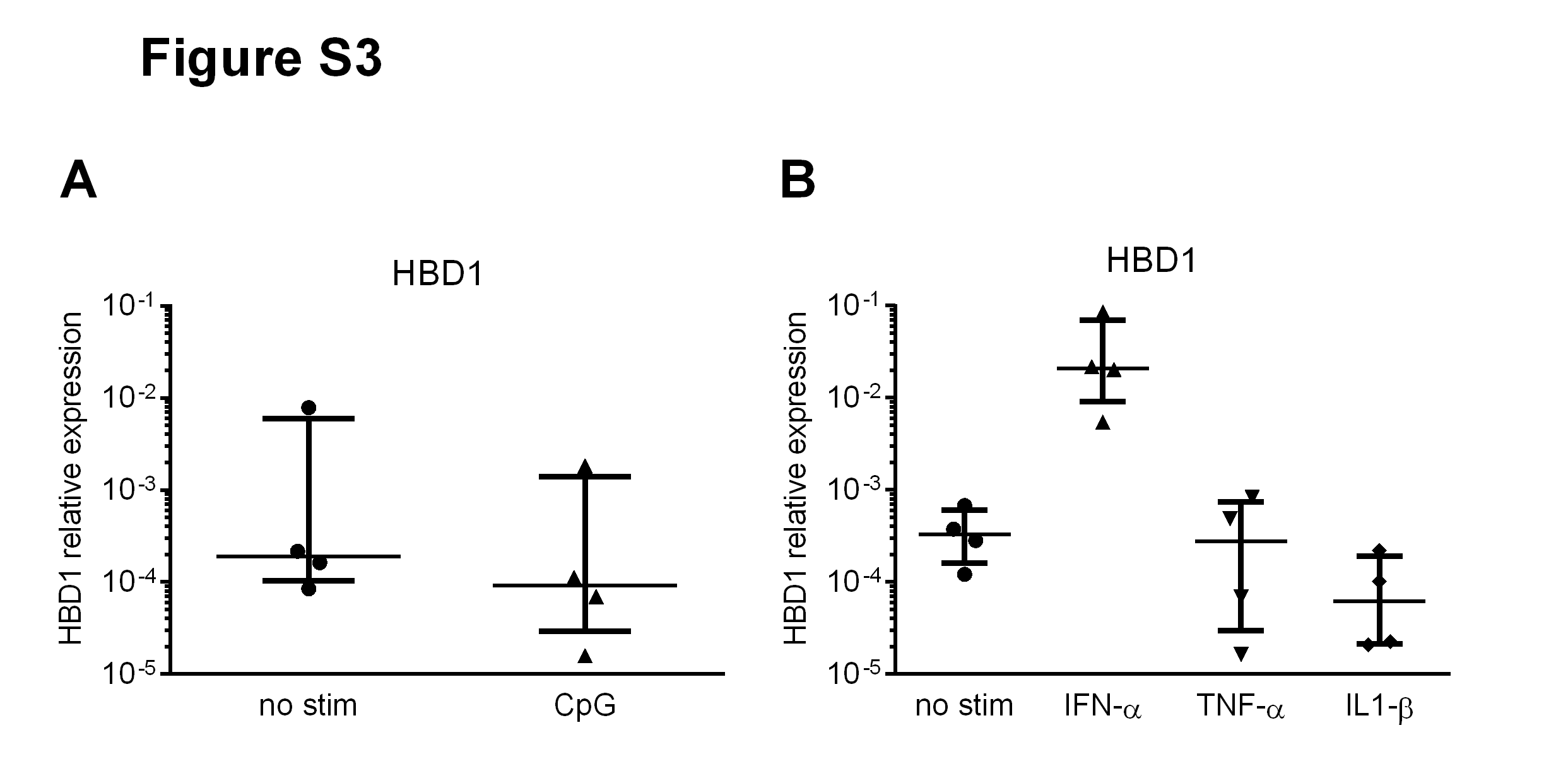

Supplement: S3 Fig — (A-C) CD14+ monocytes (n = 4) isolated using Miltenyi MACS technology were incubated with 5μM (TLR9 ligand), 10pg/ml TNF-α, 10pg/ml IL1-β or left untreated. After 24 h cells were harvested and RNA extracted. Relative expression of HBD1 was assessed using quantitative PCR. HBD1 expression was not significantly increased in monocytes when incubated with the TLR9 ligand, TNF- α or IL1-β ((A) Mann-Whitney test, (B) Kruskal-Wallis and Dunn’s multiple post comparison test). Data points are presented with median and interquartile range which each dot representing an independent experiment from a different healthy control subject. (TIF) [file pone.0173161.s003.tif]
