# Supplementary material for: Early type I Interferon response induces upregulation of human β-defensin 1 during acute HIV-1 infection
Source: PLoS One. 2017 Mar 2;12(3):e0173161. doi: 10.1371/journal.pone.0173161 (PMC5333889; doi:10.1371/journal.pone.0173161)
Supplement: S1 Table — (DOCX) [file pone.0173161.s004.docx]

**S1 Table**. Clinical and demographic data of HIV-1 infected and HIV-1 uninfected subjects

| **cohort** | **ID** | **age** | **gender** | **CD4+ T cells in**  **cells/mm^3^** | **HIV-1 viral load in**  **copies/mL** | **Years since HIV diagnosis** | **Treatment (years)** | **Co-infections**  **(self-reported)** |
| --- | --- | --- | --- | --- | --- | --- | --- | --- |
| HIV acute | \| AC015**§** \| \| --- \| \| AC018**§** \| \| AC036**§** \| \| AC040**§** \| \| AC064**§** \| \| AC084**§** \| \| AC132**§** \| \| AC202**§** \| \| AC224 \| \| AC284 \| \| AC271 \| \| AC058 \| \| AC247 \| \| AC014 \| \| AC259 \| \| AC237 \| \| AC104 \| \| AC206 \| \| AC073 \| \| AC112 \| \| AC126 \| \| AC138 \| \| AC149 \| \| AC228 \| \| AC123 \| \| AC024 \| \| AC267 \| \| AC273 \| \| **AC034** \| \| **AC329** \| \| **AC296** \| \| **AC297** \| | \| 41 \| \| --- \| \| 38 \| \| 42 \| \| 40 \| \| 33 \| \| 36 \| \| 29 \| \| 35 \| \| n/a \| \| 32 \| \| 47 \| \| 59 \| \| 43 \| \| 45 \| \| 25 \| \| 53 \| \| 36 \| \| n/a \| \| 53 \| \| 35 \| \| 34 \| \| 43 \| \| 25 \| \| n/a \| \| 31 \| \| 30 \| \| 24 \| \| 45 \| \| 44 \| \| 25 \| \| 33 \| \| 36 \| | \| male \| \| --- \| \| male \| \| male \| \| male \| \| male \| \| male \| \| male \| \| male \| \| male \| \| male \| \| male \| \| male \| \| male \| \| male \| \| male \| \| male \| \| male \| \| male \| \| male \| \| male \| \| male \| \| male \| \| male \| \| male \| \| male \| \| male \| \| male \| \| male \| \| male \| \| male \| \| male \| \| male \| | \| 413 \| \| --- \| \| 539 \| \| 399 \| \| 383 \| \| 223 \| \| 412 \| \| 815 \| \| 557 \| \| 245 \| \| 576 \| \| 397 \| \| 148 \| \| 406 \| \| 981 \| \| 714 \| \| 645 \| \| 503 \| \| 275 \| \| 508 \| \| 910 \| \| 993 \| \| 322 \| \| 510 \| \| 554 \| \| 165 \| \| 247 \| \| 324 \| \| 350 \| \| 775 \| \| 597 \| \| 395 \| \| 550 \| | \| 1490000 \| \| --- \| \| 750001 \| \| 63700 \| \| 276000 \| \| 7180000 \| \| 24700 \| \| 290856 \| \| 102000 \| \| 1000001 \| \| 2360000 \| \| 664000 \| \| 1310000 \| \| 548000 \| \| 95100 \| \| 3130 \| \| 484710 \| \| 621000 \| \| 190000 \| \| 560000 \| \| 304000 \| \| 123000 \| \| 5000000 \| \| 4100000 \| \| 1000001 \| \| 8970000 \| \| 597000 \| \| 7640000 \| \| 124000 \| \| n/a \| \| 22300000 \| \| 706000 \| \| 168000 \| | \| 0.1 \| \| --- \| \| 0.1 \| \| 0.05 \| \| 0.05 \| \| 0.05 \| \| 0.1 \| \| 0.05 \| \| 0.05 \| \| 0.2 \| \| 0.1 \| \| 0.2 \| \| 0.2 \| \| 0.2 \| \| 0.2 \| \| 0.2 \| \| 0.2 \| \| 0.2 \| \| 0.2 \| \| 0.025 \| \| 0.025 \| \| 0.025 \| \| 0.025 \| \| 0.025 \| \| 0.025 \| \| 0.05 \| \| 0.1 \| \| 0.1 \| \| 0.2 \| \| 0.2 \| \| 0.2 \| \| 0.2 \| \| 0.2 \| | \| 0 \| \| --- \| \| 0 \| \| 0 \| \| 0 \| \| 0 \| \| 0 \| \| 0 \| \| 0 \| \| 0 \| \| 0 \| \| 0 \| \| 0 \| \| 0 \| \| 0 \| \| 0 \| \| 0 \| \| 0 \| \| 0 \| \| 0 \| \| 0 \| \| 0 \| \| 0 \| \| 0 \| \| 0 \| \| 0 \| \| 0 \| \| 0 \| \| 0 \| \| 0 \| \| 0 \| \| 0 \| \| 0 \| | \| 0 \| \| --- \| \| 0 \| \| n/a \| \| 0 \| \| 0 \| \| n/a \| \| 0 \| \| 0 \| \| n/a \| \| n/a \| \| n/a \| \| HCV \| \| n/a \| \| 0 \| \| 0 \| \| 0 \| \| n/a \| \| 0 \| \| 0 \| \| 0 \| \| 0 \| \| 0 \| \| 0 \| \| n/a \| \| 0 \| \| CMV \| \| CMV , EBV \| \| 0 \| \| 0 \| \| 0 \| \| 0 \| \| 0 \| |
|  | **Total**  **32** | **Median**  **36**  **IQR**  **(32-44)** | **Male**  **100%**  **(32)** | **Median**  **458**  **IQR**  **(331-592)** | **Median**  **597000**  **IQR**  **(168000-**  **1490000)** | **Median**  **0.1**  **IQR**  **(0.05-0.2)** | **0** | **9%**  **(3)** |
| negative | \| **966971§** \| \| --- \| \| 875812**§** \| \| 427341**§** \| \| 457386**§** \| \| 471607 \| \| 773092**§** \| \| 243118**§** \| \| 334026**§** \| \| 666259**§** \| \| 991589**§** \| \| **961360** \| \| **588311** \| \| **729593** \| \| **911594** \| \| 211578 \| \| 453548 \| \| 470588 \| | \| 58 \| \| --- \| \| 39 \| \| 24 \| \| 42 \| \| 26 \| \| 24 \| \| 26 \| \| 25 \| \| 31 \| \| 29 \| \| 50 \| \| 33 \| \| 49 \| \| 47 \| \| 51 \| \| 49 \| \| 45 \| | \| male \| \| --- \| \| male \| \| male \| \| female \| \| female \| \| male \| \| female \| \| male \| \| male \| \| male \| \| male \| \| male \| \| male \| \| male \| \| male \| \| male \| \| male \| | \| n/a \| \| --- \| \| n/a \| \| n/a \| \| n/a \| \| n/a \| \| n/a \| \| n/a \| \| n/a \| \| n/a \| \| n/a \| \| n/a \| \| n/a \| \| n/a \| \| n/a \| \| n/a \| \| n/a \| \| n/a \| | \| n/a \| \| --- \| \| n/a \| \| n/a \| \| n/a \| \| n/a \| \| n/a \| \| n/a \| \| n/a \| \| n/a \| \| n/a \| \| n/a \| \| n/a \| \| n/a \| \| n/a \| \| n/a \| \| n/a \| \| n/a \| | \| n/a \| \| --- \| \| n/a \| \| n/a \| \| n/a \| \| n/a \| \| n/a \| \| n/a \| \| n/a \| \| n/a \| \| n/a \| \| n/a \| \| n/a \| \| n/a \| \| n/a \| \| n/a \| \| n/a \| \| n/a \| | \| n/a \| \| --- \| \| n/a \| \| n/a \| \| n/a \| \| n/a \| \| n/a \| \| n/a \| \| n/a \| \| n/a \| \| n/a \| \| n/a \| \| n/a \| \| n/a \| \| n/a \| \| n/a \| \| n/a \| \| n/a \| | \| n/a \| \| --- \| \| n/a \| \| n/a \| \| n/a \| \| n/a \| \| n/a \| \| n/a \| \| n/a \| \| n/a \| \| n/a \| \| n/a \| \| n/a \| \| n/a \| \| n/a \| \| n/a \| \| n/a \| \| n/a \| |
|  | **Total**  **17** | **Median**  **39**  **IQR**  **(26-49)**  **p= 0.99** | **Male**  **82%**  **(14)**  **p=0.068** | **n/a** | **n/a** | **n/a** |  | **n/a** |
| HIV progressor | \| **505402** \| \| --- \| \| **992175** \| \| **995725** \| \| 350103 \| \| 604772 \| \| **526318** \| \| 614225 \| \| 629356 \| | \| 37 \| \| --- \| \| 48 \| \| 46 \| \| 35 \| \| 63 \| \| 50 \| \| 62 \| \| 48 \| | \| male \| \| --- \| \| male \| \| male \| \| female \| \| male \| \| male \| \| male \| \| male \| | \| 803 \| \| --- \| \| 546 \| \| 773 \| \| 850 \| \| 466 \| \| 789 \| \| 453 \| \| 888 \| | \| 39795 \| \| --- \| \| 5930 \| \| 79200 \| \| 4180 \| \| 5500 \| \| 2700 \| \| 47 \| \| 48 \| | \| 12 \| \| --- \| \| 22 \| \| 3 \| \| 7 \| \| 27 \| \| 16 \| \| n/a \| \| 6 \| | \| 0 \| \| --- \| \| 0 \| \| 0 \| \| 0 \| \| 0 \| \| 0 \| \| 4 \| \| 3 \| | \| 0 \| \| --- \| \| 0 \| \| 0 \| \| 0 \| \| 0 \| \| 0 \| \| 0 \| \| 0 \| |
|  | **Total**  **8** | **Median**  **48**  **IQR**  **(39-59)**  **p=0.052** | **Male**  **88%**  **(7)**  **p=0.45** | **Median**  **781**  **IQR**  **(486-838)**  **p=0.086** | **Median**  **2959**  **IQR**  **(104-31329)**  **p=0.0007** | **Median**  **12**  **IQR**  **(6-22)** |  | **0%**  **(0)** |
| HIV controller | \| **595424** \| \| --- \| \| **828656** \| \| **271998** \| \| **588800** \| \| **550081** \| \| **275432** \| \| **330183** \| \| **931995** \| \| **562770** \| | \| 46 \| \| --- \| \| 44 \| \| 51 \| \| 46 \| \| 46 \| \| 57 \| \| 41 \| \| 41 \| \| 50 \| | \| male \| \| --- \| \| male \| \| female \| \| male \| \| male \| \| male \| \| male \| \| male \| \| male \| | \| 549 \| \| --- \| \| 1018 \| \| 1493 \| \| 624 \| \| 1167 \| \| 1050 \| \| 602 \| \| 396 \| \| 789 \| | \| 24 \| \| --- \| \| 164 \| \| 48 \| \| 177 \| \| 128 \| \| 48 \| \| 797 \| \| 48 \| \| 270 \| | \| 24 \| \| --- \| \| 13 \| \| 17 \| \| 24 \| \| 12 \| \| 25 \| \| 4 \| \| 7 \| \| n/a \| | \| 0 \| \| --- \| \| 0 \| \| 0 \| \| 0 \| \| 0 \| \| 0 \| \| 0 \| \| 0 \| \| 0 \| | \| HCV \| \| --- \| \| GBV-C \| \| HCV \| \| 0 \| \| 0 \| \| HBV \| \| 0 \| \| 0 \| \| 0 \| |
|  | **Total**  **9** | **Median**  **46**  **IQR**  **(42-51)**  **p= 0.068** | **89%**  **(8)**  **p=0.49** | **Median**  **789**  **IQR**  **(576-1109)**  **p= 0.001** | **Median**  **128**  **IQR**  **(48-224)**  **p= 0.0001** | **Median**  **15**  **IQR**  **(8.25-24)** |  | **44%**  **(4)** |

§= samples used in Figure 2 C and D; Sample ID’s in **bold**: samples used in Figure 1 C and D; p-values for age, viral load and CD4 count were calculated by Kruskal-Wallis and Dunn’s multiple comparison test compared to acute samples group; p values for gender were calculated by Chi square test compared to acute samples group.
